# Strong Antibacterial Properties of Cotton Fabrics Coated with Ceria Nanoparticles under High-Power Ultrasound

**DOI:** 10.3390/nano11102704

**Published:** 2021-10-13

**Authors:** Anna V. Abramova, Vladimir O. Abramov, Igor S. Fedulov, Alexander E. Baranchikov, Daniil A. Kozlov, Varvara O. Veselova, Svetlana V. Kameneva, Vladimir K. Ivanov, Giancarlo Cravotto

**Affiliations:** 1 Kurnakov Institute of General and Inorganic Chemistry, Russian Academy of Sciences, Leninsky Prospekt 31, 119991 Moscow, Russia; novita@mail.ru (V.O.A.); if345@ya.ru (I.S.F.); a.baranchikov@yandex.ru (A.E.B.); danilko_zlov@mail.ru (D.A.K.); ibvarvara@yandex.ru (V.O.V.); kamenevasvetlana@gmail.com (S.V.K.); van@igic.ras.ru (V.K.I.); 2 Dipartimento di Scienza e Tecnologia del Farmaco, University of Turin, Via P. Giuria 9, 10125 Turin, Italy; giancarlo.cravotto@unito.it; 3 World-Class Research Center “Digital Biodesign and Personalized Healthcare”, Sechenov First Moscow State Medical University, 8 Trubetskaya, 119991 Moscow, Russia

**Keywords:** high-power ultrasound, cavitation, finite element modeling method, fabric, nanomaterials, antibacterial properties

## Abstract

Flexible materials, such as fabric, paper and plastic, with nanoscale particles that possess antimicrobial properties have a significant potential for the use in the healthcare sector and many other areas. The development of new antimicrobial coating formulations is an urgent topic, as such materials could reduce the risk of infection in hospitals and everyday life. To select the optimal composition, a comprehensive analysis that takes into account all the advantages and disadvantages in each specific case must be performed. In this study, we obtained an antimicrobial textile with a 100% suppression of E. coli on its surface. These CeO_2_ nanocoatings exhibit low toxicity, are easy to manufacture and have a high level of antimicrobial properties even at very low CeO_2_ concentrations. High-power ultrasonic treatment was used to coat the surface of cotton fabric with CeO_2_ nanoparticles.

## 1. Introduction

Recently, there has been a noticeable increase in bacterial resistance to medical drugs, which, in turn, leads to an intensive search for antimicrobial agents [1]. Nanomaterials have a very high ratio of particle surface area to volume and, as a result, have unique physical and chemical properties and are highly effective antimicrobial agents [2]. In this regard, nanomaterials have the ability to inhibit the growth of microorganisms and can play an essential role in antimicrobial therapy. Interestingly, nanoparticles can easily be deposited onto the surfaces of textiles, which causes the materials to acquire bactericidal properties. Silver nanoparticles are often used for this purpose [1,2] but are very expensive. Metal oxides, such ZnO, TiO_2_, CuO and MgO, show high antimicrobial activity and may be an alternative to silver nanoparticles [3,4,5]. Recently, nanoscale CeO_2_ particles have demonstrated promising antimicrobial activity, as well [6,7]. There are numerous publications that describe the antimicrobial properties of such nanoscale oxides [5,6,7,8,9,10,11,12,13].

However, there is no widespread use of antibacterial textiles to date. This is due to the fact that there is no industrial technology that can produce antimicrobial textiles on a large scale and at a low price. Furthermore, the composition of such a coating should ideally be effective against a wide range of bacteria and fungi.

Back in the 1970s, it was first shown that cerium nitrate has a powerful antiseptic effect in the treatment of burn wounds, especially in the treatment of Gram-negative bacteria and fungi [14]. In the case of cerium compounds applied to bandages, no patients developed necrotic infections, resulting in a 50% reduction in the mortality rate. Nanodisperse cerium dioxide is another promising material that is widely used in modern high-tech industries including biomedical applications. Low toxicity of nanodisperse cerium dioxide [15] provides enough safety when using it in vivo, which makes it possible to consider this material as a promising drug for the treatment of a number of diseases [16,17]. 

Until now, there is no scalable method for the application of cerium oxide nanoparticles on textile materials. The objective of this work is a comparative analysis of the antibacterial properties of coatings made of CeO_2_ and ZnO nanoparticles that were deposited onto the surface of cotton fabrics. These metal-oxide-modified fabrics were prepared under similar conditions using high-power ultrasonic treatment. This technique is a proven method for the application of nanoparticles onto the surface of woven and nonwoven materials that can be scaled and applied industrially [3]. The comparative analysis of antibacterial characteristics of CeO_2_- and ZnO-modified fabrics will enable us to evaluate the potential of the widespread use of CeO_2_-coated textiles.

## 2. Materials and Methods

The following methods were used to prepare the ceria-containing sols for the obtaining of antibacterial coatings:

### 2.1. CeO_2_ Sol Synthesis

The procedure of CeO_2_ sol hydrothermal synthesis from ammonium hexanitratocerate (NH_4_)_2_Ce(NO_3_)_6_ has been described in detail by Shcherbakov et al. [18]. First, 2.33 g of (NH_4_)_2_Ce(NO_3_)_6_ was dissolved in 23 mL of distilled water and placed in a glass autoclave. The hydrothermal synthesis was carried out at 95 °C for 24 h. The resulting light-yellow precipitate was separated by centrifugation, washed three times with isopropanol and redispersed in 25 mL of deionized water. To remove the residual isopropanol, the solution was boiled for 1 h with the addition of deionized water to maintain the original volume. The CeO_2_ concentration in the resulting yellow sol was determined gravimetrically.

### 2.2. TEMPO-Oxidized Cellulose Synthesis

A “blue ribbon” filter paper (LLC Melior XXI, Moscow, Russia) was used as a source of cellulose. We hydrolyzed 4 g of filter paper with an 18% hydrochloric acid solution at 80 °C for 40 min. The resulting dispersion of nanocrystalline cellulose was washed with distilled water to pH = 5. To introduce carboxyl groups into the polymer, nanocrystalline cellulose was modified via TEMPO oxidation [19,20]. The volume of the dispersion of nanocrystalline cellulose was adjusted to 400 mL with distilled water; then 0.4 g of sodium bromide and 0.067 g of TEMPO were added. Oxidation was carried out via the dropwise addition of 50 mL of 11.9% NaClO solution under vigorous stirring. The pH value of the reaction mixture was maintained at 10–10.5. At the end of the synthesis, the mixture was stirred for 1 h, and the pH was then adjusted to neutral with a 0.1 M hydrochloric acid solution. The resulting precipitate of TEMPO-oxidized nanocrystalline cellulose was washed with distilled water, separated by centrifugation and placed in 60 mL of distilled water. The modified cellulose suspension was purified using dialysis for 3 days. The purified cellulose suspension was ultrasonically dispersed at 16–19 °C for 2 h. The concentration of TEMPO-oxidized nanocrystalline cellulose in the dispersion was determined gravimetrically. The concentration of carboxyl groups in the obtained cellulose samples (0.8 mmol per gram of cellulose) was measured via conductometric titration.

### 2.3. CeO_2_/Cellulose Sol Synthesis

To obtain CeO_2_ sol modified with cellulose, equal volumes of the initial ligand-free CeO_2_ sol (concentration of CeO_2_ 10 g/L) and the dispersion of TEMPO-oxidized nanocrystalline cellulose (concentration 4.7 g/L) were mixed under vigorous stirring (the molar ratio of CeO_2_ to the monomer unit of cellulose is 1/0.5). To obtain control samples, a dispersion of TEMPO-oxidized nanocrystalline cellulose, with a concentration of 4.7 g/L, was used.

### 2.4. Coating of the Textile

The samples were submerged in the ceria-containing sols and treated with high-power ultrasound to immobilize the cerium oxide onto the textile surface. Samples were cut from a prewashed fabric. The dimensions of the samples were 10 × 10 cm. The cut-out samples were dried in a drying cabinet at a temperature of 70 °C for 1 h. Next, the samples were fixed on a frame that was placed in the center of the reaction vessel, which was filled with the appropriate solution at a temperature of 20 °C. The samples were treated with ultrasound at a frequency of 22 kHz, which was delivered to the vessel using a waveguide system that was connected to a magnetostrictive ultrasonic emitter. The emitter was powered by an ultrasonic generator. The maximum power of the generator was 1000 W.

**Figure 1:**
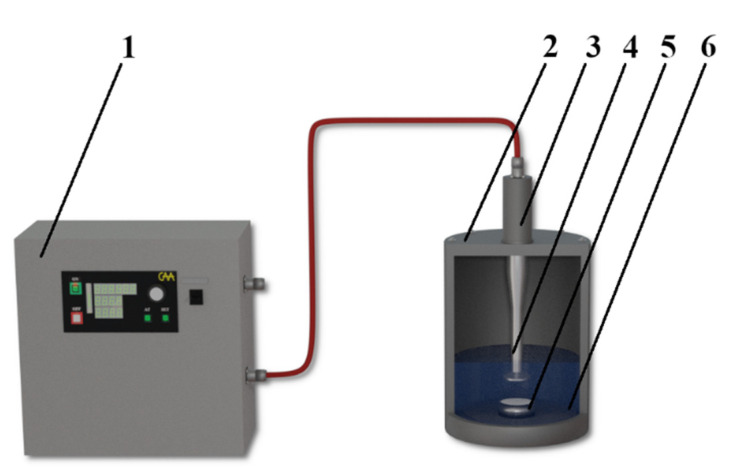
Experimental setup for coating the fabric surface with nanoscale particles: 1—ultrasonic generator; 2—treatment vessel; 3—magnetostrictive transducer; 4—waveguide system; 5—a special frame with fixed fabric sample; 6—suspension of nanoparticles.

The scheme of the experimental setup is shown in Figure 1. The distance between the ultrasonic emitter and the frame was 20 mm. Control samples were obtained by soaking cotton fabrics in sols of similar concentration and composition, followed by washing and drying. To determine the effect of ultrasonic treatment on the fixation of sol particles on the surface of cotton fabrics, some samples were washed in a stream of water. Cotton fabric samples were fixed on a special frame. 

### 2.5. Modeling of Acoustic Fields

**Figure 2:**
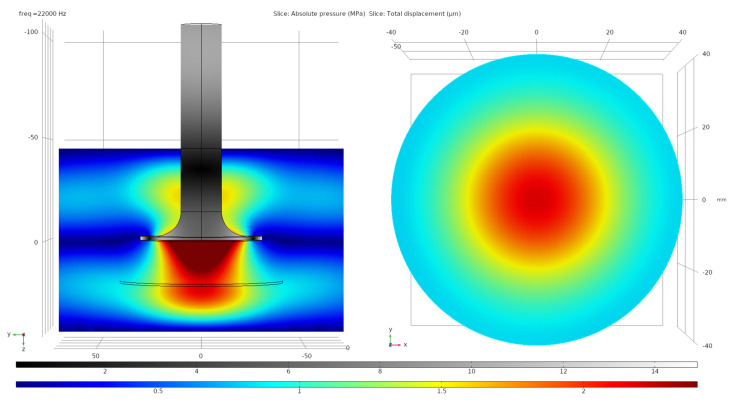
Calculations of the distribution of the amplitudes of longitudinal vibrations in the waveguide system and the acoustic pressures in the reaction vessel and on the surface of the processed cotton fabric.

We modeled the oscillations of the transducers and developed waveguides using the COMSOL Multiphysics Software (version 5.6, Solid Mechanics software module), which is a software that uses the finite element method. During the equipment design, we calculated the dimensions of the elements in such a way that their own mechanical resonance frequency coincides with the resonance of the magnetostrictive transducer. At the second step of calculations, the acoustics module of the software was used. It allowed us to calculate and use special functions for acoustic field visualization. These calculations were carried out under the assumptions that there is no cavitation and that the woven material absorbs 50% of the ultrasound. The results of the calculations of the acoustic fields in the vessel for the ultrasonic processing of samples are shown in Figure 2. 

The calculations showed that the distribution of acoustic fields is uniform in the first approximation inside a circle with a diameter of 40 mm located at a distance of 20 mm from the surface of the waveguide system. Therefore, for the research, we cut out round samples with a diameter of 40 mm. In this case, the distribution of nanoparticles on their surface was homogeneous.

### 2.6. Antibacterial Activity Test

To check the level of antibacterial properties of the obtained coatings, a suspension of *E. coli* K12 cells with an optical cell density of OD = 0.8 was used. The fabric samples were placed in the culture medium, LB + 1.5% agar. They were subsequently coated with 100 μL of cells. After 12 h of incubation at 37 °C, the fabric samples were removed from the medium, and the residual antibacterial effect was tested on Petri dishes as incubation continued at 37 °C. The studies of the antibacterial properties were conducted in the dark.

The quantitative characteristics of the antibacterial activity of textile materials were determined according to the standard method [21].

The antibacterial activity, *A*, of the samples was determined using the formula: *A* = *F* − *G*, where *F* is the bacterial growth rate on the control samples (log10 CFU/mL after incubation – log10 CFU/mL prior to incubation), and *G* is the bacterial growth rate on the test samples. During the experiments, the decimal logarithm of the colony-forming units was calculated, which is an indicator of the number of viable microorganisms per unit volume. In samples with a conditional antibacterial activity of 10, the complete suppression of bacteria was observed (no colonies were observed after incubation).

### 2.7. Materials Characterization

X-ray diffraction analysis was performed using a diffractometer D8 Advance (Bruker, Billerica, MA, USA) with a CuKα_1,2_ source and rotating anode diffractometer Rigaku D/MAX 2500 (Rigaku, Tokyo, Japan), using CuKα_1,2_ radiation and a graphite monochromator. The diffraction maxima were identified using the ICDD database. The full-profile analysis of diffractograms was performed using the Le Bail method and the free software package Jana2006 [22]. The size of the crystallites was estimated using the Scherrer equation. The degree of cellulose crystallinity was evaluated using the Segel method, with the intensity of the <200> (*I*_200_) reflex and the minimum between the <110> and <200> reflexes corresponding to the signal of the amorphous (*I_AM_*) phase [23]:

*CI* = (*I*_200_ − *I_AM_*)/(*I*_200_) × 100%

IR spectra were measured on a Fourier transform spectrometer FSM 2202 (Infraspek, St. Petersburg, Russia) in the range from 400 to 4000 cm^−1^ with a resolution of 2 cm^−1^. Conductometric titration was performed using the Expert 002 (Econix, Moscow, Russia) conductometer.

Scanning electron microscopy (SEM) and energy-dispersive X-ray spectroscopy (EDS) were performed on NVision 40 and LEO SUPRA 50 VP (Zeiss, Oberkochen, Germany) microscopes equipped with X-MAX energy-dispersive detectors (Oxford Inst., Abingdon, UK) at accelerating voltages of 10 and 20 kV. Transmission electron microscopy (TEM) analysis was performed using an electron microscope Libra 200 MC (Zeiss, Oberkochen, Germany) at an accelerating voltage of 200 kV.

The amount of cerium oxide deposited onto the surface of the cotton fabric was determined thermogravimetrically. To do this, pieces of cotton fabric were placed in a corundum crucible before and, after ultrasonic treatment, heated at a rate of 1 °C/min and annealed at 800 °C for 3 h. The surface concentration of CeO_2_ in the studied samples was calculated on the basis of the measured weight loss of the initial cotton fabric and after the immobilization of cerium oxide. 

## 3. Results

**Figure 3:**
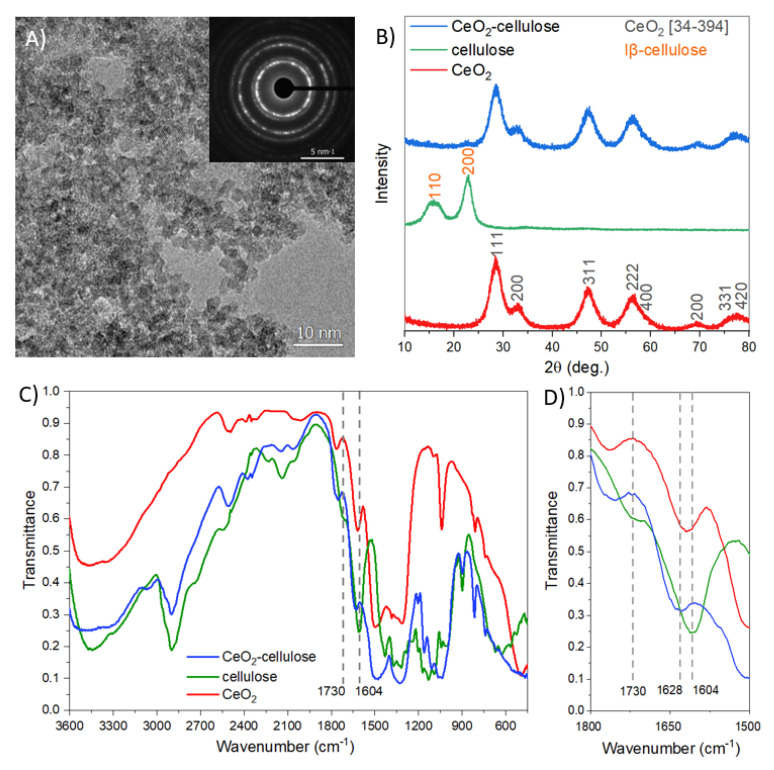
TEM image of initial CeO_2_ nanoparticles and the corresponding electron diffraction pattern (insert) (**A**), X-ray diffraction patterns (**B**), survey (**C**) and detailed (**D**) FTIR spectra of CeO_2_ nanoparticles, cellulose and CeO_2_/cellulose composite.

We have studied CeO_2_ sols used for deposition onto the surface of cotton fabrics in an ultrasonic field in detail. Based on the transmission electron microscopy image (Figure 3A) and electron diffraction pattern (Figure 3A, insert), the obtained sol contains nanocrystalline cerium oxide particles with sizes of 3.5 ± 0.6 nm (Figure 3A). X-ray diffraction (Figure 3B) indicates that both the initial CeO_2_ sol and sol modified with TEMPO-oxidized cellulose comprise crystalline cerium oxide (ICDD 34–0394). The size of ceria nanoparticles calculated using the Scherrer equation is about 4 nm, which is in a good agreement with the TEM results. The XRD pattern of initial TEMPO-oxidized nanocrystalline cellulose contains <110> and <200> reflections of type Iβ cellulose at 2θ = 16° and 23° [24]. The crystallinity of cellulose is 76%, as determined using the Segal method. In the CeO_2_/cellulose XRD pattern, a low intensity diffraction maximum corresponding to the <200> reflection of type Iβ cellulose is observed.

FTIR spectroscopy of nanocrystalline cellulose (Figure 3C) shows a broad absorption band in the range of 3500–3100 cm^−1^ that relates to the stretching vibrations of the –OH groups; absorption bands of stretching vibrations of –CH and –CH_2_ at 2900 cm^−1^; stretching vibrations of C=O at 1650 cm^−1^; bending vibrations of CH_2_ at 1430 cm^−1^; and a broad absorption band of stretching vibrations of C–O–C and CO at 1200–1000 cm^−1^ [24,25]. After oxidation using TEMPO, additional absorption bands at 1730 and 1604 cm^−1^ appear in the spectrum of nanocrystalline cellulose, and these bands correspond to the vibrations of the carboxyl group in the protonated and nonprotonated forms, respectively [26]. The FTIR spectra of the powders obtained by drying the CeO_2_ and CeO_2_/cellulose sols show the absorption bands of the stretching vibrations of CeO_2_ in the range of 500–700 cm^−1^ [27,28], whereas the absorption bands at 1040 and 830–810 cm^−1^ are probably related to nitrate ions adsorbed onto the nanocrystalline ceria surface [28]. The absorption bands of OH groups adsorbed onto the CeO_2_ surface are at 3400 and 1620 cm^−1^ [28]. The FTIR spectrum of the CeO_2_/cellulose powder demonstrates a new complex absorption band with a maximum at 1628 cm^–1^. The position of this band is notably shifted with respect to the corresponding band of bare TEMPO-oxidized cellulose (1604 cm^–1^) containing deprotonated carboxylic groups, which are coordinated with Na^+^ ions. Such a shift reflects the changes in coordination mode of –COO^–^ groups, indicating their interaction with positively charged [18] CeO_2_ nanoparticles. 

**Figure 4:**
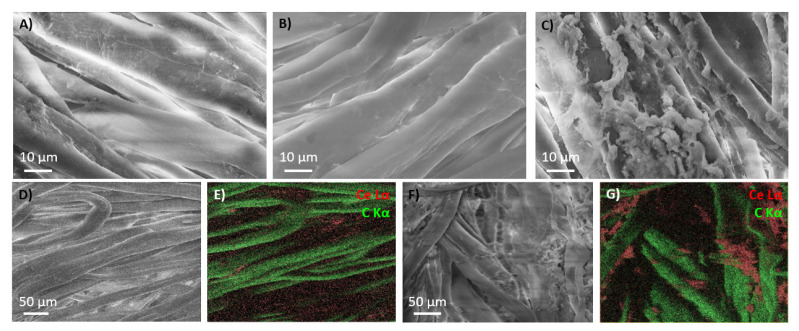
SEM images of the textile after ultrasonic treatment in the cellulose dispersion (**A**), the nonmodified CeO_2_ sol (**B**) and the CeO_2_/cellulose sol (**C**). SEM images and the corresponding cerium- and carbon-distribution EDS maps of the textile after ultrasonic treatment in nonmodified CeO_2_ sol (**D**,**E**) and CeO_2_/cellulose sol (**F**,**G**).

The microstructure of the textile samples obtained using ultrasonic treatment in sols of nonmodified CeO_2_ and cellulose-bound cerium oxide was studied using scanning electron microscopy (Figure 4). The morphology of the textile fibers after ultrasonic treatment in both ceria and cellulose sols does not differ from the original (Figure 4A,B). Furthermore, according to EDS mapping (Figure 4D,E), ultrasonic treatment results in a regular ceria distribution on the textile surface when using pure CeO_2_ sol. On the other hand, the ultrasonic treatment of the textile using ceria that was modified with TEMPO-oxidized cellulose leads to the aggregation of CeO_2_/cellulose on the textile surface (Figure 4C). Based on EDS mapping, these agglomerates consist mostly of ceria (Figure 4F,G). Ultrasonic treatment causes the hydrogen bonds on the surfaces of both CeO_2_ and the cellulose nanoparticles to rearrange [29]. Presumably, ceria facilitates the TEMPO-oxidized cellulose sol–gel transition upon sonication due to the formation of new hydrogen bonds and, probably, coordination with carboxylic groups. The obtained nonhomogenous hydrogel absorbed onto the textile samples forms the ceria-rich agglomerates after drying.

**Figure 5:**
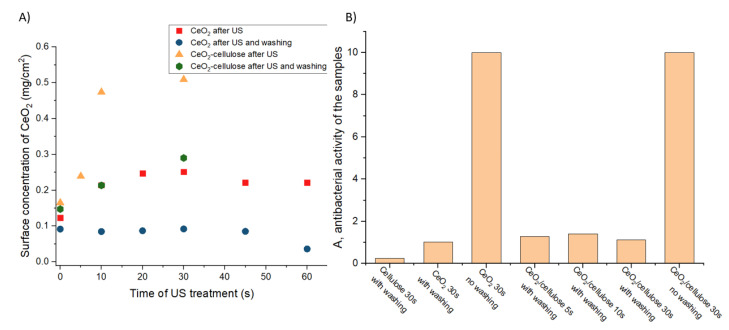
(**A**) The dependence of the CeO_2_ concentration on the surface of the cotton fabric on ultrasonic treatment time. (**B**) Antibacterial activity of the samples.

It is very important to reduce the treatment time when working on an industrial scale. We therefore studied the effect of the treatment duration on the concentration of the nanoparticles on the surface of the textile material. In a series of experiments, ultrasonic treatment was carried out for 0–60 s using both the nonmodified CeO_2_ sol and the sol in which CeO_2_ was mixed with cellulose (Figure 5A). A sample that was obtained via impregnation in the corresponding sols for 30 s was used for comparison. 

Comparison of the concentrations of cerium oxide on the textile surface after ultrasonic treatment in various sols, with and without subsequent washing, showed that the presence of cellulose significantly increased the surface concentration of ceria. In the case of the nonmodified ceria sol, the concentration of CeO_2_ only slightly differs from the sample obtained via impregnation. Although washing leads to a significant decrease in the CeO_2_ concentration in all samples, relative concentrations are still larger in the CeO_2_/cellulose samples.

The samples coated with CeO_2_ and with CeO_2_/cellulose both demonstrated extremely high antibacterial activity after preparation (full suppression of *E. coli*) (Figure 5B). It should be noted that the double membrane of E. coli, a widespread Gram-negative bacterium, provides good protection against several types of antibiotics, making E. coli one of the six bacteria types that are commonly spread in hospitals. The antibacterial activity of the textile samples against this type of bacteria may indicate the potential of the coating against other Gram-negative bacteria, such as salmonella and legionella.

The obtained results confirmed that it is possible to use ultrasound to apply CeO_2_ nanoparticles onto the surface of textiles. These coated fibers may be used in bandages and other medical textiles, which are usually single-use. The high antibacterial activity of CeO_2_-coated textiles indicates the high potential of the material.

We also carried out analyses of the resistance of the coating to washing. It is important to understand whether textile materials coated with CeO_2_ using ultrasonic oscillations are feasible for hospital gowns, which are reused after washing. However, a very large decrease in antibacterial properties was observed after washing of both types of samples (Figure 5B). Although, upon washing, the concentration of ceria in the CeO_2_/cellulose samples was higher than in the CeO_2_ samples (see Figure 5A), the antibacterial activity of both of these samples was virtually equal. It was therefore not possible to obtain samples that would retain their properties during washing. The reason for that could be the weakness of the chemical connection between CeO_2_ and cellulose.

In order to understand the efficiency of the developed technology, we have compared the obtained results with the antibacterial properties of cotton fabrics coated with ZnO using the same technique. The coating of the surface of cotton fabrics with ZnO nanoparticles was carried out according to the protocol described elsewhere [8]. It should be noted that the studies of the antibacterial properties of both types of coatings were carried out simultaneously under identical conditions. The results of the comparative experiments are presented in Table 1.

The antibacterial properties, in the initial state, of the samples that were coated with CeO_2_ and the samples coated with CeO_2_/cellulose were higher than in the samples with the ZnO coating. After washing, the antimicrobial properties of the samples coated with ZnO were significantly higher than those of both the samples coated with CeO_2_ and CeO_2_/cellulose. A possible explanation for this is the more powerful physical linkage of the larger particles of ZnO with cellulose. However, increasing the size of CeO_2_ particles may lead to an increase in cytotoxicity. Thus, the use of antibacterial cotton fabrics with the nano-CeO_2_ coating is only possible in products intended for a single use, while cotton fabrics coated with ZnO should be used in products that will be washed.

## 4. Conclusions

We have developed an ultrasonic technique for coating the surface of cotton fabrics with CeO_2_ and CeO_2_/cellulose nanoparticles in order to provide them with antimicrobial properties.

The ultrasonic treatment results in a regular distribution of ceria on the textile surface when pure CeO_2_ sol is used. On the other hand, the ultrasonic treatment of the textile using ceria that was modified with cellulose leads to the aggregation of CeO_2_/cellulose on the textile surface. 

The samples of cotton fabric coated with nanoparticles of CeO_2_ and CeO_2_/cellulose demonstrated excellent antimicrobial activity in the experiments with E. coli conducted in the dark. The antibacterial properties measured under similar conditions for the samples coated with CeO_2_ nanoparticles were higher than for the samples coated with ZnO.

Washing the coated samples resulted in a very significant decrease in the concentration of CeO_2_ particles on the surface of the cotton fabric, while, for samples with cellulose, the nanoparticles were better retained on the surface. However, for both types of CeO_2_-containing samples, a very significant decrease in antibacterial activity was observed after washing. At the same time, the antibacterial properties of the cotton fabrics coated with ZnO nanoparticles and washed under the same conditions changed only slightly.

In contrast to ZnO, CeO_2_ nanoparticles were easily washed off, and the antimicrobial activity of the material noticeably decreased.

The conducted experiments indicate that the nanoparticles of CeO_2_ are promising for applications in disposable dressings but that they cannot be used to cover medical textiles that are subjected to washing.

The developed ultrasonic processes are quite fast, do not require significant capital expenditures upon scale up and are able to ensure the formation of a uniform antibacterial nanocoating.

## Figures and Tables

**Table 1:** Antibacterial properties of cotton fabrics with nanoscale particle coatings.

Type of Coating	Antimicrobial Activity *A*, Initial State	Antimicrobial Activity *A*, after Washing
CeO_2_	No colonies were observed after incubation	1.02
CeO_2_/cellulose	No colonies were observed after incubation	1.11
ZnO	4.5	3.3

## Data Availability

Data presented in this article are available on request from the corresponding author.
